# Immediate Free Jejunum Transfer for Salvage Surgery of Gastric Tube Necrosis

**DOI:** 10.1155/2014/327549

**Published:** 2014-10-01

**Authors:** Hiroki Umezawa, Takeshi Matsutani, Rei Ogawa, Hiko Hyakusoku

**Affiliations:** ^1^Department of Plastic, Reconstructive and Aesthetic Surgery, Nippon Medical School, 1-1-5 Sendagi, Bunkyo-ku, Tokyo 113-8603, Japan; ^2^Department of Gastrointestinal and Hepato-Biliary-Pancreatic Surgery, Nippon Medical School, 1-1-5 Sendagi, Bunkyo-ku, Tokyo 113-8603, Japan

## Abstract

Gastric tube necrosis after esophagus cancer surgery is a rare but critical situation. Salvage reconstruction of the esophagus remains a challenging procedure for head and neck surgeons. Historically, surgeons have employed a two-stage salvage surgery consisting of debridement followed by reconstruction. While this procedure generates good results, the time to restart oral alimentation is long. The present report describes the case of a 62-year-old male who developed gastric tube necrosis 3 days after undergoing surgery for thoracic-cervical esophageal cancer and immediate reconstruction with the retrosternal gastric pullup technique. He was treated with debridement and simultaneous free jejunum transfer 4 days after the primary surgery. He was able to restart oral alimentation 10 days after the salvage surgery. This rapid return to oral alimentation is a major advantage of the one-stage immediate esophagus salvage reconstruction. Another advantage is the ease of the reconstructive procedure: the absence of scarring and prolonged inflammation, which are disadvantages of the two-stage procedure, meant that recipient vessel selection and anastomosis were uncomplicated. The one-step procedure may be particularly useful in cases where the inflammation is discovered early.

## 1. Introduction

Reconstructing esophageal defects has been challenging for reconstructive surgeons, but gastric pullup and colon interposition have become common procedures for total esophageal replacement. However, partial necrosis at the oral side of the flap, fistula formation, or stricture sometimes occurs chiefly because of insufficient blood supply or because of too much tension at the anastomosis. In these cases, secondary surgery should be considered. In the past, two-stage surgery was commonly performed for this purpose. The first step was the removal of necrotic tissue and the second step was reconstruction. The interval between these two steps ranged from 1 to 6 months [1]. The outcomes of this treatment are acceptable and patients can start oral ingestion on average 14 days after the reconstruction step.

The reason for delaying the reconstruction step is concern that immediate reconstruction after debridement could induce intense inflammation and expose the patient to serious infections such as mediastinitis. However, there are also advantages to immediate reconstruction after debridement. First, the recipient vessels will generally be in good condition because there is minimal inflammation around the vessels. Second, the patient can start oral ingestion much earlier [2].

In this report, the case of a patient who underwent simultaneous debridement and esophagus salvage reconstruction 4 days after the primary surgery is described. The recipient vessel was still in good condition and further inflammation did not occur.

## 2. Case Report

**Figure 1:**
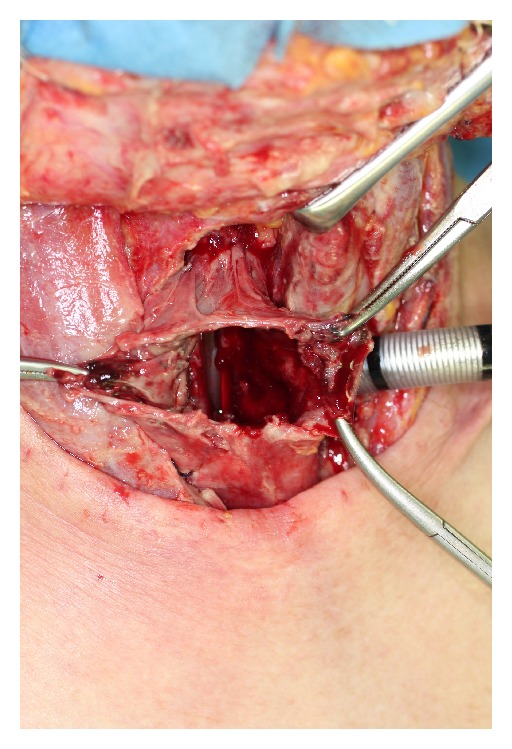
The gastric tube was necrotic and had ruptured.

**Figure 2:**
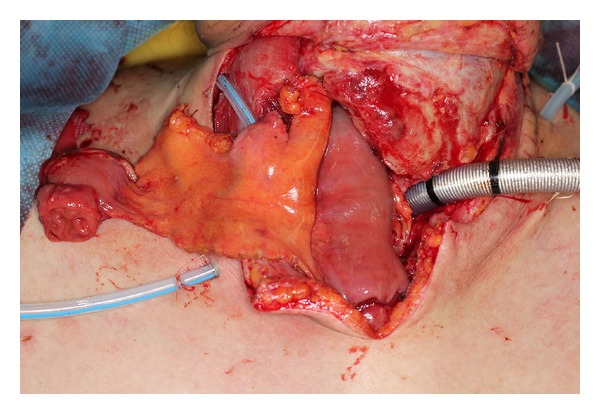
The necrotic tissue was debrided; the route of the gastric tube was changed from retrosternal to presternal, and immediate free jejunum transfer was performed.

**Figure 3:**
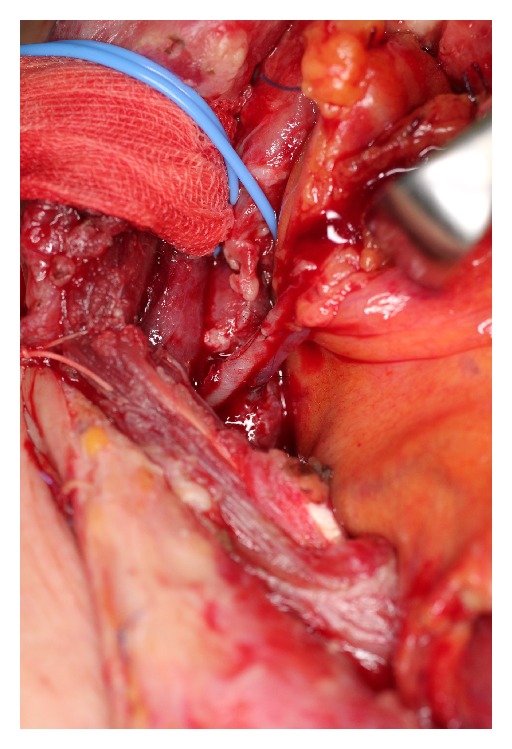
The jejunal artery and vein were anastomosed to the right transverse cervical artery and the right internal jugular vein, respectively.

**Figure 4:**
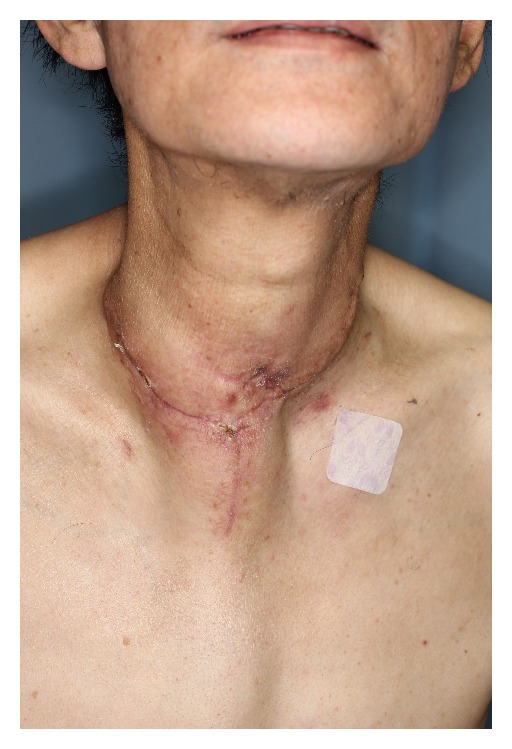
One month after the salvage surgery. The wound had healed completely.

A 62-year-old male underwent surgery for thoracic-cervical esophageal cancer and immediate reconstruction with the retrosternal gastric pull-up technique. The esophagus resection was performed at the level of the 6th cervical vertebra and a bilateral lower segmental neck dissection was performed (nos. 101 to 104) [3]. Therefore, the transverse cervical vessel was exposed in first surgery. Three days after surgery, the patient's neck exhibited swelling and redness, and the oral side of the pulled-up stomach became necrotic (Figure 1). Thus, 4 days after the primary surgery, the patient underwent debridement of the necrotic tissue and the route of the gastric tube was changed from retrosternal to presternal. The resection of the necrotic gastric tube was 5 cm length, and debridement of necrotic tissue and washing with saline solution were performed. At the same time, immediate free jejunum transfer was performed as salvage surgery (Figure 2). The jejunum was transected at the level of the second jejunal vessel, and the length of harvesting jejunum graft was 15 cm. After trimming of the jejunum graft, the jejunum graft, which was 10 cm length, was placed in the presternal space and joined to the distal end of the cervical esophagus and the proximal end of the residual gastric tube in an end to end anastomosis. The jejunal artery and vein were anastomosed to the right transverse cervical artery and the right internal jugular vein (Figure 3). Because of the short span between first and second surgery, harvesting the free jejunum graft was performed without any difficulty. However, neck dissection during the first surgery caused some adhesion, which resulted in minor technical difficulties during microsurgery. In second time surgery, patient's vital signs were stable, and no fever or problems in respiratory or circulatory organs were observed. The C-reactive protein level was 22.6 mg/dL, but the white blood cell level was 8.7 × 10³/*μ*L. The patient was able to start oral alimentation 10 days after salvage surgery, and the wound healed completely without complications (Figure 4). Although he died from systemic metastasis from the primary cancer 6 months after the salvage surgery, the patient's quality of life after surgery, including his food intake, was very good.

## 3. Discussion

The advantages of the one-stage immediate esophageal salvage reconstruction surgery are the avoidance of recipient vessel inflammation and an early return to oral ingestion. In addition, fresh vascularized tissue transfer is expected to prevent more severe inflammation and infection such as mediastinitis or sepsis [2]. However, the disadvantages of one-stage immediate esophageal salvage reconstruction surgery include the fact that it may be difficult to determine the resection margin of necrotic tissue. This is problematic because incomplete debridement could result in severe infection; moreover, the remaining inflammation could result in vascular anastomosis thrombosis [4]. Large clinical trials that examine the frequency of such poor outcomes in one-stage immediate esophageal salvage reconstruction surgery are warranted.

Reports of two-stage esophageal salvage reconstruction surgery [1, 5, 6] show that the reconstruction surgery step is generally performed after a relatively long time, 1–6 months, after debridement. This delay relates to the inflammation remaining after debridement, which must subside before reconstruction can be performed [1, 5, 6].The advantage of this approach is its safety in terms of infection. However, its disadvantage is that the recipient vessels needed for the reconstruction may be damaged by the scarring arising from the debridement and the prolonged inflammation, which complicates recipient vessel selection. In addition, the scars can impede the transferred intestinal tube anastomosis and gastrointestinal motility. Moreover, this approach can be complicated by incomplete debridement or infection around the fistula of pharynx or esophagus, both of which further delay the timing of the salvage reconstructive surgery and the restart of ingestion.

Thus, when performing one-stage immediate esophagus salvage reconstruction, it is necessary to identify and completely resect the necrotic tissues. One should also consider whether to change the intestinal route of reconstruction. Appropriate antibiotic administration and systematic infection control should also be performed. In particular, it is essential that inflammation is discovered early. Indeed, we believe that an indication of the one-stage salvage reconstruction is the early discovery of gastric tube necrosis. The benefits of one-stage immediate esophagus salvage reconstruction include the easy reconstruction procedure and the rapidity with which the patient's quality of life is improved.
